# Entropy–Based Diversification Approach for Bio–Computing Methods

**DOI:** 10.3390/e24091293

**Published:** 2022-09-14

**Authors:** Rodrigo Olivares, Ricardo Soto, Broderick Crawford, Fabián Riquelme, Roberto Munoz, Víctor Ríos, Rodrigo Cabrera, Carlos Castro

**Affiliations:** 1 Escuela de Ingeniería Informática, Universidad de Valparaíso, Valparaíso 2362905, Chile; 2 Escuela de Ingeniería Informática, Pontificia Universidad Católica de Valparaíso, Valparaíso 2362807, Chile; 3 Departamento de Informática, Universidad Técnica Federico Santa María, Valparaíso 2390123, Chile

**Keywords:** Shannon entropy, bio–computing methods, improved global search, multidimensional knapsack problem

## Abstract

Nature–inspired computing is a promising field of artificial intelligence. This area is mainly devoted to designing computational models based on natural phenomena to address complex problems. Nature provides a rich source of inspiration for designing smart procedures capable of becoming powerful algorithms. Many of these procedures have been successfully developed to treat optimization problems, with impressive results. Nonetheless, for these algorithms to reach their maximum performance, a proper balance between the intensification and the diversification phases is required. The intensification generates a local solution around the best solution by exploiting a promising region. Diversification is responsible for finding new solutions when the main procedure is trapped in a local region. This procedure is usually carryout by non-deterministic fundamentals that do not necessarily provide the expected results. Here, we encounter the stagnation problem, which describes a scenario where the search for the optimum solution stalls before discovering a globally optimal solution. In this work, we propose an efficient technique for detecting and leaving local optimum regions based on Shannon entropy. This component can measure the uncertainty level of the observations taken from random variables. We employ this principle on three well–known population–based bio–inspired optimization algorithms: particle swarm optimization, bat optimization, and black hole algorithm. The proposal’s performance is evidenced by solving twenty of the most challenging instances of the multidimensional knapsack problem. Computational results show that the proposed exploration approach is a legitimate alternative to manage the diversification of solutions since the improved techniques can generate a better distribution of the optimal values found. The best results are with the bat method, where in all instances, the enhanced solver with the Shannon exploration strategy works better than its native version. For the other two bio-inspired algorithms, the proposal operates significantly better in over 70% of instances.

## 1. Introduction

In computer science and mathematical optimization, nature–inspired methods are considered higher–level heuristics designed to find or generate potential solutions or to select a heuristic (partial search algorithm). These methods may provide near–optimal solutions in a limited amount of time. They properly work with incomplete or imperfect information or with bounded computation capacity [1]. These metaheuristic algorithms are inspired by interesting natural phenomena, such as the species’ selection and evolution mechanisms [2], swarm intelligence like the pathfinding skills of ants [3] and the attraction capabilities of fireflies [4], or the echolocation behavior of microbats [5]. Even physical [6] and chemical [7] laws have also been studied to design metaheuristic methods. During the last two decades, metaheuristics have attracted the scientific community’s attention due to their versatility and efficient performance when adapted to intractable optimization problems [8]. Different metaphors have guided the design of uncountable metaheuristic methods [9]. By grouping the metaheuristic algorithms according to their inspiration source, it is possible to detect at least the bio–inspired computation class, swarm intelligence methods, and genetic evolution. In this context, the literature has shown that when these techniques come from similar analogies, they often share common behavioral patterns, mainly in the initial parameter task and the intensification and diversification processes [10].

The evolutionary strategy of these bio–inspired techniques mainly depends on the appropriate balance between the diversification and the intensification phases. Diversification or exploration is the mechanism of entirely visiting new points of a search space. Intensification or exploitation is the process of refining those points within the neighborhood of previously visited locations to improve their solution quality [11]. When the diversification phase operates, the resolution process reduces accuracy to improve its capacity to generate new potential solutions. On the other hand, the intensification strategy allows refining existing solutions while adversely driving the process to locally optimal solutions.

Although metaheuristic algorithms present outstanding performance [12], they suffer from a common problem that arises when the exploration and exploitation process must be balanced, even more so when the number of variables in the problem increases. The greater the number of variables to handle, the more iterations will be necessary to find the best solution, thus converging the search in a specific area of the solution space [13]. In this context, all solutions are similar and can be considered good quality. Therefore, the iterative process that tries to improve existing solutions stagnates as it cannot continue to improve without leaving the feasible zone [1]. Different external methods have been used to solve this problem, such as random walk [14,15], roulette wheel [16,17,18], tabu list [19,20], among others. These mechanics allow the solution to be modified to move it from that space area to another. This movement alters the solutions randomly. The non–deterministic behavior that governs the update procedures lacks information to discriminate when to operate and which part of the region to visit.

In this work, we propose an efficient exploration module for bio–inspired algorithms based on Shannon entropy, a mathematical component to measure the average level of uncertainty inherent in observations from random variables [21,22,23]. The objective is to detect stagnation in local optimum, through a predictive entropy system, by computing entropy values of each variable. Next, solutions are moved toward a feasible region to find new and better solutions. This proposal is implemented and evaluated in three well–known population–based metaheuristics: particle swarm optimization, black hole algorithm, and bat optimization. The choice of these metaheuristics is supported by: (a) they work similarly because they belong to the same type of algorithms based on the swarm intelligence paradigm; (b) these population–based metaheuristics describe an iterative procedural structure to evolve their individuals (solutions), followed by many bio–inspired optimization algorithms; and (c) they have proven to be efficient optimization solvers for complex engineering problems. However, hybrid techniques such as [24,25] are also welcome. The Shannon diversification strategy runs as a background process and does not have an invasive role in the principal method.

Finally, to evidence that the proposed approach is a viable alternative that improves bio-inspired search algorithms, we evaluate it on a set of the most challenging instances of the Multidimensional Knapsack Problem (MKP), which is a widely recognized NP–complete optimization problem [26]. MKP was selected because it is suitable for Shannon’s diversification strategy, it has a wide range of practical applications [27], and it continues to be a hot topic in the operations research community [28,29]. Computational experiments run on 20 of the most challenging instances of the MKP taken from OR–Library [30]. Generated results are evaluated with descriptive analysis and statistical inference, mainly a hypothesis contrast by applying non–parametric evaluations.

The rest of this manuscript is structured as follows. Section 2 discusses the bibliographic search for relevant works in the field, fundamental concepts related to the diversification and the intensification phases, and it describes the information theory to measure uncertainty levels in random variables. Section 3 exposes the formal statement for the stagnation problem. Section 4 presents the developed solution, including the main aspects of the three bio–computing algorithms and the integration with the Shannon entropy. In Section 5, the experimental setup is detailed, while Section 5 discusses the main obtained results. Finally, conclusions and future work are included in Section 8.

## 2. Related Work

During the last two decades, bio-inspired computing methods have attracted the scientific community’s attention due to their remarkable ability to adapt search strategies to solve complex problems [31]. They are considered solvers devoted to tackling large instances of complex optimization problems [32,33]. These algorithms can be grouped according to their classification. Here, we can observe a division into nature-inspired vs. non-nature-inspired, population-based vs. single point search—or single solution—, dynamic vs. static objective function, single neighborhood vs. various neighborhood structures, and memory usage vs. memory-less methods, among many others [34,35,36].

Metaheuristics can usually provide near–optimal solutions in a limited time when no efficient problem–specific algorithm pre–exists [32]. After studying several metaheuristics, we can state that they operate similarly by combining local improvement procedures with higher-level strategies to explore the space of potential solutions efficiently [37,38,39]. During the last decades, metaheuristic algorithms have been analyzed by finding improved techniques capable of solving complex optimization problems [40]. This evolution has enabled them to merge theoretical principles from other science fields. For instance, Shannon entropy [21,22,23] has been used in a population distribution strategy based on historical information [41]. The study reveals a close relationship between the solutions’ diversity and the algorithm’s convergence. Also, ref. [42] proposed a multi–objective version of the particle swarm optimization algorithm enhanced by the Shannon entropy. The authors propose an evolution index of the algorithm to measure its convergence. Results show that the proposal is a viable alternative to boost swarm intelligence methods, even in mono–objective procedures. Another work that deals with metaheuristics enhanced by the Shannon entropy to treat multi–objective problems is [43]. Here, the uncertain information was employed to choose the optimum solution from the Pareto front. Shannon’s strategy was slightly lower than other proposed decision–making techniques.

Now, by considering smart alterations in search processes, we analyze [44], which proposes an entropy–assisted particle swarm optimizer for solving various instances of an optimization problem. This approach allows for adjusting the exploitation and exploration phases simultaneously. The reported computational experiments show that this work provides flexibility to the bio–inspired solver to self–organize its inner behaviors. Following this line of research, in [45], a hybrid algorithm between Shannon entropy and two swarm methods is introduced to improve the yield, memory, velocity, and, consequently, the move update. In [46], Shannon entropy is integrated into a chaotic genetic algorithm for taking data from solutions generated during the execution. This process runs in deterministic time series and operates from the initial population strategy. Another work that develops a similar proposal is detailed in [47]; here, the authors present a hybrid algorithm that includes the Shannon entropy in the evolving process of particle swarm optimization. The authors measure the convergence of solutions based on the distance between each solution and the best overall solution. They conclude that the algorithm can satisfactorily obtain outstanding results, especially regarding fitness evolution and convergence rate.

Following the integration between bio–inspired solvers and the Shannon entropy, we analyze [48], where the information component allows measuring the population diversity, the crossover probability, and the mutation operator to adjust the algorithm’s parameters adaptively. Results show that it is possible to generate a satisfactory global exploration, improve convergence speed, and maintain the algorithm’s robustness. The same approach is explored in [49]. Again, the convergence speed and the population diversity are key factors, balanced to improve the resolution procedure. Finally, in [50,51], the Shannon entropy allows handling the instance of the optimization problem. The first work solves the portfolio selection problem by minimizing the number of transactions, while the second computes the minimum loss and cost of the reactive power planning.

## 3. Preliminaries

This section briefly defines Shannon entropy and explains the stagnation problem.

### 3.1. Shannon Entropy

Claude Elwood Shannon proposed a mathematical component capable of measuring an information source’s uncertainty level based on the probability distribution followed by the data that compose it [22]. This component is called entropy, and it applies to different fields. In computer science, it is known as information entropy and is expressed by the following formula [22,23]:

(1) $$H\left(x\right)=-\sum_{i=1}^{n}p\left(x\right)\log_{2}p(x)$$

where x corresponds to a possible system event, and p(x) represents the probability that the event *x* occurs. The entropy of an information system is calculated from a set of possible events occurring in the system, together with their occurrence probability. The formula returns values between 0 to +∞, corresponding to the entropy level of the evaluated system. Results close to 0 mean low entropy, so the system is considered more predictable. On the contrary, the higher the result, the higher the entropy, and the system is considered less predictable.

### 3.2. Stagnation Problem

Mono–objective optimization problems are commonly modeled as follows:

(2) $$\begin{array}{c} \arg\min_{\vec{x}}\;f\left(\vec{x}\right)\\ \mathrm{subject to}\\ g_{i}\left(\vec{x}\right)\leq 0,\;\forall \;i=\left\{1,2,\ldots ,m\right\}\\ h_{j}\left(\vec{x}\right)=0,\;\forall \;j=\left\{1,2,\ldots ,p\right\} \end{array}$$

where $\vec{x}=\langle x_{1},x_{2},\ldots ,x_{n}\rangle$ is an *n*-dimensional vector that represents the set of decision variables—or solutions—, $f(\vec{x})$ is the function to be optimized—or objective function—, and both $g_{i}\left(\vec{x}\right)$ and $h_{j}\left(\vec{x}\right)$ are the constraint sets of the problem.

The fitness is the output value obtained from evaluating a solution in the objective function. In a constrained optimization problem, the aim is to find the best fitness among several solutions which satisfy all constraints. Over the decades, many techniques have been developed to solve complex optimization problems. Recently, bio–inspired computing methods—or metaheuristics—have emerged as solvers able to generate fitness near to optimal values [9]. These nature–inspired mechanisms mimic the behavior of individuals in their environment based on survival of the fittest. This strategy allows altering solutions into new solutions that are potentially better than previous ones. The update is carried out by interactive search procedures, such as exploration and exploitation [52]. The exploration phase focuses on discovering new zones of non–visited heuristic spaces. The exploitation phase intensifies the local search process in an already visited promising zone. In both cases, the evolutionary operators are mathematical formulas abstracted from the natural phenomenon that inspires the algorithm, invoked for random time lapses.

Despite the outstanding performance of bio–inspired algorithms in solving complex problems, they present a common problem: local optima stagnation. The stagnation problem is formally defined as the premature convergence of an algorithm to a solution that is not necessarily the best or close to it [53]. Widely proven techniques treat this topic using internal characteristics, such as the memory of movements, prohibitive elements, and random restart, among several other methods. These procedures generally operate probabilistically, randomly alter solutions, and do not use the knowledge generated in the resolution process [52,53]. This issue presents an interesting problem: *How much can we improve an algorithm if it internally considers the knowledge generated to recognize local optima stagnation and escape from it during the search process?* Currently, the information produced by the optimization algorithms is not analyzed due to its uncertain and random nature. Nevertheless, in the next section, we will see that this is possible and useful for this problem.

## 4. Developed Solution

In this section, we detail how the Shannon entropy is applied to detect the stagnation issue on bio–computing methods and how it is also employed to escape from this problem.

### 4.1. Bio–Inspired Methods

We employ three population–based metaheuristic algorithms: particle swarm optimization, black hole algorithm, and bat optimization. These algorithms are among the most popular swarm intelligence methods. The first one is inspired by the behavior of birds flocking or fish schooling when they move from one place to another. The second one is based on the black hole phenomenon when it attracts and absorbs stars of a constellation. The third one imitates the echolocation phenomenon present in the microbats species, allowing them to avoid obstacles while flying and locate food or shelter. In general terms, the three metaheuristics work similarly. For example, solution positions are randomly generated at the beginning of the algorithm and updated via an alteration of the velocity.

#### 4.1.1. Particle Swarm Optimization

In particle swarm optimization (PSO), each bird or fish describes a particle—or solution—with two components: position and velocity. A set of particles (the candidate solutions) forms the swarm that evolves during an iterative process giving rise to a powerful optimization method [31]. The method works by altering velocity through the search space and then updating its position according to its own experience and that of neighboring particles.

The traditional particle swarm optimization is governed by two vectors, the velocity ${\vec{v}}_{i}=\langle v_{i}^{1},v_{i}^{2},\ldots ,v_{i}^{j},\ldots ,v_{i}^{n}\rangle$ and the position ${\vec{x}}_{i}=\langle x_{i}^{1},x_{i}^{2},\ldots ,x_{i}^{j},\ldots ,x_{i}^{n}\rangle$. First, the particles are randomly positioned in an *n*-dimensional heuristic space with random velocity values. During the evolution process, each particle updates its velocity and position—see Equation (3) and Equation (4), respectively—:

(3) $$v_{i}^{j}\left(t+1\right)=wv_{i}^{j}\left(t\right)+c_{1}\phi_{1}\left(pBest_{i}^{j}-x_{i}^{j}\left(t\right)\right)+c_{2}\phi_{2}\left(gBest^{j}-x_{i}^{j}\left(t\right)\right)$$

(4) $$x_{i}^{j}\left(t+1\right)=x_{i}^{j}\left(t\right)+v_{i}^{j}\left(t+1\right)$$

where *w*, $c_{1}$, and $c_{2}$ are acceleration coefficients to obtain the new velocity, $\phi_{1}$ and $\phi_{2}$ are uniformly distributed random values in the range [0,1], $pBest_{i}$ represents the previous best position of *i*th particle, and finally, gBest is the global best position found by all particles during the resolution process.

#### 4.1.2. Black Hole Algorithm

The black hole (BH) algorithm begins with randomly generated initial positions of stars ${\vec{x}}_{i}=\langle x_{i}^{1},x_{i}^{2},\ldots ,x_{i}^{j},\ldots ,x_{i}^{n}\rangle$, each with a velocity of change ${\vec{v}}_{i}=\langle v_{i}^{1},v_{i}^{2},\ldots ,v_{i}^{j},\ldots ,v_{i}^{n}\rangle$. Both vectors belong to an *n*-dimensional heuristic space for an optimization problem.

The best candidate is chosen at each iteration to become in the black hole. At this moment, other solutions are pulling around the black hole, building the constellation of stars. A star will be permanently absorbed when it gets too close to the black hole. A new solution is randomly generated and located in the search space to keep the number of stars balanced. The absorption of stars by the black hole is formulated in Equations (5) and (6):

(5) $$v_{i}^{j}\left(t+1\right)=r\left(bh^{j}-x_{i}^{j}\left(t\right)\right)$$

(6) $$x_{i}^{j}\left(t+1\right)=x_{i}^{j}\left(t\right)+v_{i}^{j}\left(t+1\right)$$

where *r* is a uniformly distributed random value in the range [0,1], and bh represents the global best location—or black hole—found by all the stars during the resolution process.

This algorithm attempts to solve the stagnation problem using a random component that acts when a participation probability is reached. This procedure is known as the event horizon and plays an important role in controlling the global and local search. The black hole will absorb every star that crosses the event horizon. The radius of the event horizon is calculated by Equation (7):

(7) $$E=\frac{f(bh)}{\sum_{i=1}^{s}f(x_{i})}$$

where *s* represents the number of stars, f(bh) is the fitness value of the black hole, and $f(x_{i})$ is the fitness value of the *i*th star. When the distance between a *i*th star and the black hole ($diff_{i}$) is less than the event horizon at an instant *t*, the star collapses into the black hole. The distance between a star and a black hole is the Euclidean distance computed by Equation (8) as follows:

(8) $$diff_{i}\left(t\right)=\sqrt{{\left[bh^{1}-x_{i}^{1}\left(t\right)\right]}^{2}+{\left[bh^{2}-x_{i}^{2}\left(t\right)\right]}^{2}+\cdots +{\left[bh^{j}-x_{i}^{j}\left(t\right)\right]}^{2}+\cdots +{\left[bh^{n}-x_{i}^{n}\left(t\right)\right]}^{2}}$$

As mentioned above, this procedure operates under random criteria. If a real random number between 0 and 1 is higher than an input parameter, the event horizon provides diversity among solutions. Otherwise, solutions keep intensifying the current search area.

#### 4.1.3. Bat Optimization

Naturally inspired, bat optimization (BAT) follows the behavior of microbats when they communicate through bio–sonar, an inherent feature of this species. Bio–sonar, also called echolocation, allows bats to determine distances and distinguish between food, prey, and background obstacles. The algorithm extrapolates this characteristic, assuming that all bats develop it. In this abstraction, a bat flies at a position $x_{i}$ with a velocity $v_{i}$ and emitters sounds with a frequency $f_{i}$, a loudness $A_{0}$, and a pulse emission rate r∈[0,1].

Similar to the previous metaheuristics, bat optimization is driven by two vectors, the velocity ${\vec{v}}_{i}=\langle v_{i}^{1},v_{i}^{2},\ldots ,v_{i}^{j},\ldots ,v_{i}^{n}\rangle$ and the position ${\vec{x}}_{i}=\langle x_{i}^{1},x_{i}^{2},\ldots ,x_{i}^{j},\ldots ,x_{i}^{n}\rangle$. Again, both vectors belong to an *n*–dimensional heuristic space. Furthermore, the frequency varies in a range $\left[f_{min},f_{max}\right]$, and loudness can vary in many ways. It is assumed that it ranges from a large (positive) value $A_{0}$ to a minimum constant value $A_{min}$.

The algorithm starts with an initial population of bat positions. The best position is selected in each iteration according to its yield, called the global solution. To find new solutions, the frequencies is computed by Equation (9), in order to adjust the new velocity, which is updated via Equation (10) and thus, new position are generated by Equation (11).

(9) $$\begin{array}{c} f_{i}=f_{min}+\left(f_{max}-f_{min}\right)\beta \end{array}$$

(10) $$\begin{array}{c} v_{i}^{j}\left(t+1\right)=\left(x_{best}^{j}-x_{i}^{j}\left(t\right)\right)f_{i} \end{array}$$

(11) $$\begin{array}{c} x_{i}^{j}\left(t+1\right)=x_{i}^{j}\left(t\right)+v_{i}^{j}\left(t+1\right) \end{array}$$

where β is a uniformly distributed random value in the range [0,1], $f_{min}$ is set to have a small value, and $f_{max}$ varies according to the max variance allowed in each time step. Next, $x_{best}$ describes the global best solution generated by all bats during the search process.

In bat optimization, the random walk trajectory governs the diversification phase to alter a solution. The new solution is generated based on the bat’s current loudness $A_{i}$ and maximum allowed variance max(var) during a time step. This procedure is computed by Equation (12).

(12) $$\begin{array}{c} x_{new}^{j}=x_{old}^{j}+\epsilon A_{i}max(var) \end{array}$$

where ϵ is a random value in [−1,1].

Finally, the variation between loudness and pulse emission drives the intensification phase. This principle is given from hunter behavior when bats recognize prey. When it occurs, they decrease loudness and increase the rate of pulse emission. This strategy is calculated by Equation (13).

(13) $$\begin{array}{c} A_{i}=\alpha A_{i},\;r_{i}=r_{i}^{time=0}\left(1-e^{-\gamma (time=t)}\right) \end{array}$$

where α and γ are ad-hoc constants to control the intensification phase. For 0<α<1 and γ>0, we get $A_{i}\to 0$, $r_{i}\to r_{i}^{\left(time=0\right)}$, t→0.

#### 4.1.4. Common Behavior

Population–based metaheuristics work similarly. We present a common work scheme (see Algorithm 1) to implement each bio–inspired solver. This scheme adapts the generalities of the search process and the particularities of each bio-inspired phenomenon.

### 4.2. Solving Stagnation

Our proposal covers two scopes: detecting stagnation and escaping from it. For the first step, we employ historical knowledge of metaheuristics while solving optimization problems. Next, a new formula based on random work is used, which considers certain information about the algorithm’s performance.

#### 4.2.1. Stagnation Detecting

**Figure 1:**
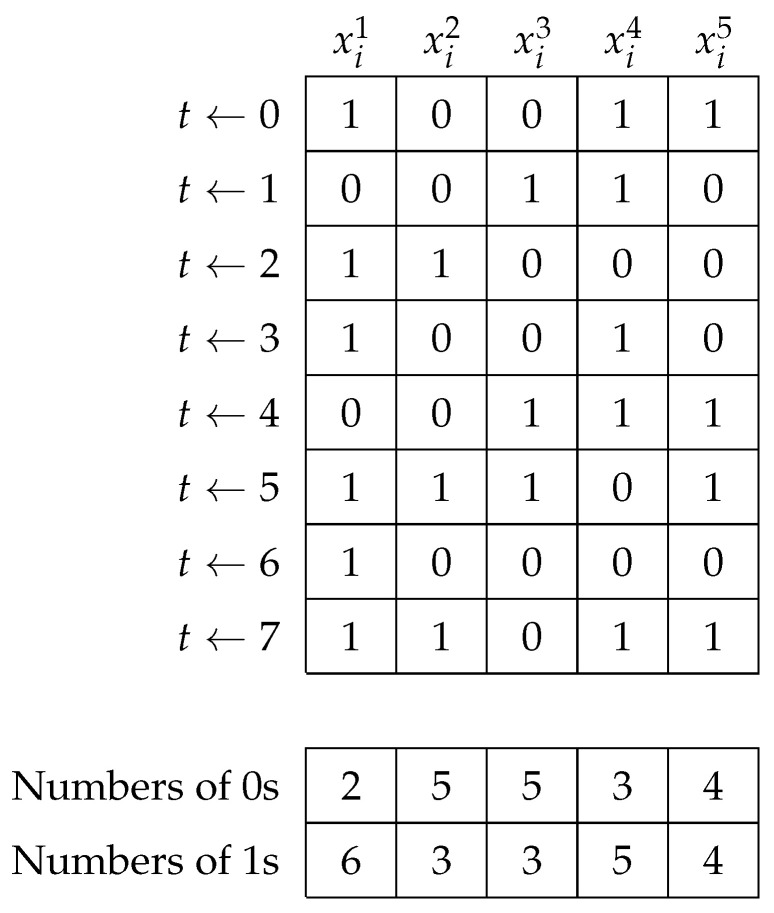
Example of history of changes from a solution vector along to iterations.

By considering a binary approach for each agent, this would remember the number of times their status changes (zero to one and one to zero) into decision variables $x_{i}^{j}$ until a time *t*. Figure 1 presents a history of changes for a solution vector of 5–dimension.
**Algorithm 1:** Common work scheme used to implement the population–based algorithms
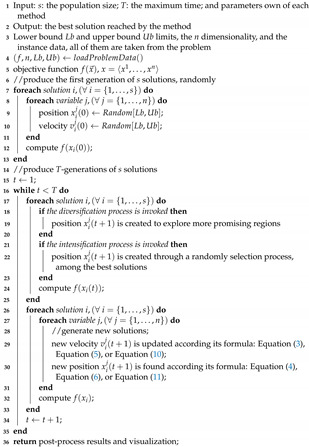


**Figure 2:**
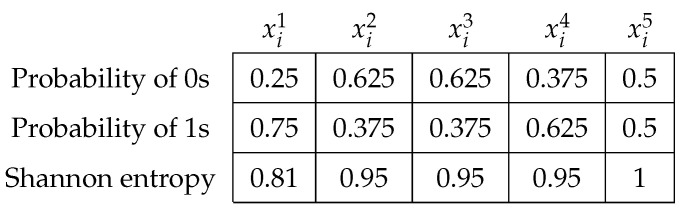
Probabilities and Shannon entropy values.

The evolutionary operator updated this solution vector seven times. It was initially generated in t←0. Next, we computed the simple probability of occurrence for all decision variables. With these values, we finally calculate and show the Shannon entropy of each one in Figure 2:

Entropy values close to 0.3 give us a trust value near 95%, i.e., when a solution vector generates this occurrence level, we will be in the presence of stagnation. It is relevant to note that the population of solutions is randomly instantiated under a uniform probability. Hence, first iterations do not produce low entropy values. For the above example, the Shannon entropy values 0.81, 0.95, 0.95, 0.95, and 1 are all far from 0.3, so the vector cannot be considered as stagnated. We consider that a vector stagnates if the median value of the Shannon entropy is at least 0.3. The procedure operation can be seen in Algorithm 2.
**Algorithm 2:** Shannon entropy module
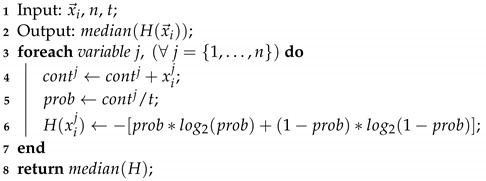


The procedure requires the current solution vector ${\vec{x}}_{i}$, the number of decision variables *n*, and the time *t* it was called. In the end, the module provides the element right in the middle of the solution vector, which means at least half of the entropies reached the trust value. For that, Lines 3–6 compute the Shannon entropy values under the occurrence probability of state changes.

From the spatial complexity point of view, the proposal uses historical data but does not store it. Regarding temporal complexity, entropy values are computed after the evolutionary operator, so the solution vector is linearly traversed at different time steps.

#### 4.2.2. Stagnation Escaping

As mentioned above, bio–inspired algorithms use random modifiers to balance the exploration and then avoid the stagnation problem. This strategy suggests that changes in decision variables have the same distribution and are independent of each other. Therefore, it assumes the past movement cannot be used to predict its future values [54,55]. Our proposal goes the opposite way, applying the entropy value in a similar form to a random walk but using historical data to make a decision. We propose to update only those decision variables that we already know a priori that are stagnant. The latter is one of the main differences in the random walk method. This modifier is formulated in Equation (14).

(14) $$\begin{array}{c} x_{new}^{j}=x_{i}^{j}+\lambda H\left(x_{i}^{j}\right) \end{array}$$

where λ is an uniformly distributed random integer value in the {−1,0,1}. A positive or negative value for λ provides to explore new promising zones. Furthermore, setting $H(x_{i}^{j})$ as a smaller step size allows the exploration phase to not stray too far from the current solution. This operation remains agnostic to bio–inspired algorithms, working on both native solver systems and hybridized/improved versions.

## 5. Experimental Setup

**Figure 3:**
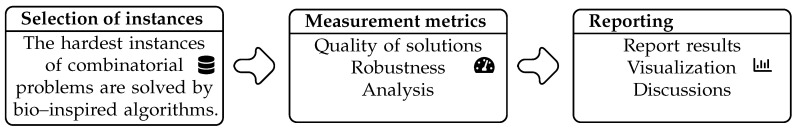
Schema of the experimental phase applied to this work.

To suitably evaluate the performance of the improved swarm intelligence methods, a robust performance analysis is required. For that, we contrast the best solutions achieved by metaheuristics to the best–known result of the benchmark. Figure 3 depicts the procedures involved in thoroughly examining the enhanced metaheuristics. We design goals and suggestions for the experimental phase to show that the proposed approach is a viable alternative for enhancing the inner mechanisms of metaheuristics. Solving time is computed to determine the produced gap when the Shannon strategy runs on the bio–inspired method. We evaluate the best value as a vital indicator for assessing future results. Next, we use ordinal analysis and statistical testing to evaluate whether a strategy is significantly better. Finally, we detail the hardware and software used to replicate computational experiments. Results will visualize in tables and graphics.

A set of optimization problem instances were solved for the experimental process and, more specifically, to measure the algorithms’ performance. These instances come from OR–Library, which J.E. Beasley originally described in 1990 [56]. This “virtual library” has several test data sets of different natures with their respective solutions. We take 20 binary instances of the Multidimensional Knapsack Problem. Instances are identified by a name and a number, in the form MKP1 to MKP20, respectively. Table 1 details the size of each instance.

The exact methods have not solved the instances from MKP17 to MKP20. For this reason, we use unknown to describe that this value has not yet been found.

The MKP is formally defined in Equation (15):

(15) $$\begin{array}{c} \max\sum_{j=1}^{n}v_{j}x_{j}\\ \mathrm{subject to}\\ \sum_{j=1}^{n}w_{jk}x_{j}\leq b_{k},\;\forall \;k\in \left\{1,\ldots ,K\right\}\\ x_{j}\in \left\{0,1\right\} \end{array}$$

where $x_{j}$ describes whether the object is included or not in a knapsack, and the *n* value defines the total number of objects. Each object has a real value $v_{j}$ that represents its profit and is used to compute the objective function. Finally, $w_{jk}$ stores the weight for each object in a knapsack *k* with maximum capacity $b_{k}$. As can be seen, this is a combinatorial problem between including or not an object. The execution of continuous metaheuristics in a binary domain requires a binarization phase after the solution vector changes [60]. A standard Sigmoid function compared to a uniform random value δ between 0 and 1 was employ as a transformation function, i.e., $[1/\left(1+e^{-x_{i}^{j}}\right)]>\delta$, then a discretization method is employed, in this case, if $x_{i}^{j}\leftarrow 1$. Otherwise, $x_{i}^{j}\leftarrow 0$.

The performance of each algorithm is evaluated after solving each instance 30 times on it. Once the complete set of outputs of all the executions and instances has been obtained, an outliers analysis is performed to study possible irregular results. Here, we detect influence outliers using the Tukey test, which takes as a reference the difference between the first quartile (Q1) and the third quartile (Q3), or the interquartile range. In our case, in box plots, an outlier is considered to be 1.5 times that distance from one of those quartiles (mild outlier) or three times that distance (extreme outlier). This test was implemented by using a spreadsheet, so the statistics are calculated automatically. Thus, we remove them to avoid distortion of the samples. Immediately, a new run is launched to replace the eliminated solution.

In the end, descriptive and statistical analyses of these results are performed. For the first one, metrics such as max and min values, mean, standard quasi–deviation, median, and interquartile range are used to compare generated results. The second analysis corresponds to statistical inference. Two hypotheses are contracted to evidence a major statistical significance: (a) test of normality with Shapiro–Wilk and (b) test of heterogeneity by Wilcoxon–Mann–Whitney. Furthermore, it is essential to note that given the independent nature of instances, the results obtained in one of them do not affect others. The repetition of an instance neither involves other repetitions of the same instance.

Finally, all algorithms were coded in Java 1.8 programming language. The infrastructure was a workstation running Windows 10 Pro operating system with eight processors i7 8700, and 32 GB of RAM. Parallel implementation was not required.

## 6. Discussion

The first results are illustrated in Table 2, which is divided into three parts: (a) number of best reached, (b) minimum solving time, and (c) maximum solving time. Results show that modified methods (S–PSO, S–BAT, and S–BH) exhibit better performance achieving greater optimum values than their native versions.

Regarding the minimum and maximum solving times, PSO and S–PSO have similar performance, and a significant difference is not appreciable, being who better yield shows of all studied techniques. When BAT and S–BAT are contrasted, we again note that the Shannon Entropy strategy does not cause a significant increase in required solving time by the bio–solver algorithm, except in a few instances where BAT needs less time than S–BAT. Now, let us compare the results generated by the black hole optimizer and the improved S–BH. It is possible to observe that, in general terms, there is no significant difference between the minimum solving times required by the original bio–inspired method and its enhanced version.

To robust the experimental phase, we evaluate the quality of solutions based on the number of optimal findings. Thus, taking the generated data, we note that the modified algorithms S–PSO, S–BAT, and S–BH perform better than their native versions. Based on the result in Table 3 regarding the solution quality, S–PSO has a better performance than PSO. The latter is because S–PSO has a smaller maximum RPD, implying that his solutions are larger. Moreover, considering the standard deviations, we can see that values achieved by PSO are usually lower than those generated by S–PSO. Therefore, the results distribution and their differentiation concerning the average is better in the algorithm based on the Shannon entropy.

The result of PSO in the median RPD is equal to or slightly lower than S–PSO. Thus, PSO has a slightly better performance. For the average RPD, both S–PSO and PSO had a similar performance. Finally, considering that the proposed approach S–PSO found a more significant number of optima, with a higher quality of solutions and a lower deviation, then we can conclude that S–PSO has a better general performance than native PSO.

The results shown in Table 4 show how S–BAT presents a much higher general performance than his native version. S–BAT has, for the 20 instances, maximum, mean, and average RPDs equal to, or less than, those presented by the native bat optimizer, in addition to having a smaller standard deviation for their values. This set of characteristics implies that the solutions found by S–BAT are significantly larger than those found by the bat algorithm, and, therefore, have a higher general performance compared to its native version.

Concerning Table 5, we can infer that S–BH algorithm has a higher general performance than the BH algorithm. There are no significant differences between both algorithms in the mean RPD and average RPD, but the maximum RPD in 18 of the 20 instances is lower in S–BH. The above means that the solutions found by S–BH have a larger size and, therefore, a better performance. On the other hand, concerning its standard deviations, in 13 of the 20 instances, the native version of the algorithm has a lower variation. Therefore, both algorithms generally present a high dispersion in their data. Since both algorithms have a similar widespread distribution, based on the number of optima found, we can conclude that the S–BH algorithm has a higher overall performance because it can find a more significant number of better quality optima.

**Figure 4:**
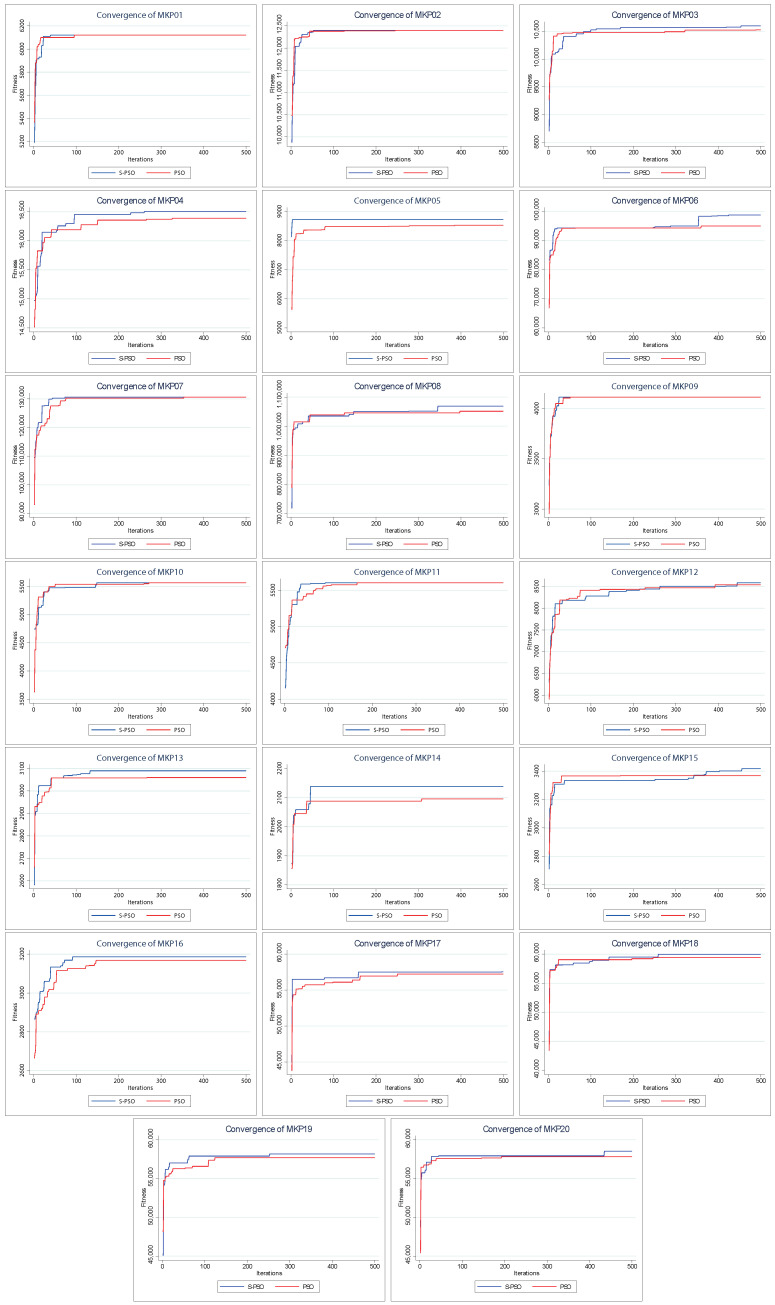
Convergence charts of PSO vs. Shannon PSO.

Figure 4 shows the convergence of the solutions found by PSO and S–PSO. The above indicates how the solution changes as the iterations go by (increases its value).

For all instances, in early iterations, S–PSO has at least a similar performance to PSO. As the execution progresses, S–PSO gradually acquires higher–value solutions. In MKP instances 2, 7, 9, 10, and 11, both algorithms have the same final performance. S–PSO is superior in all other instances (15 of 20).

**Figure 5:**
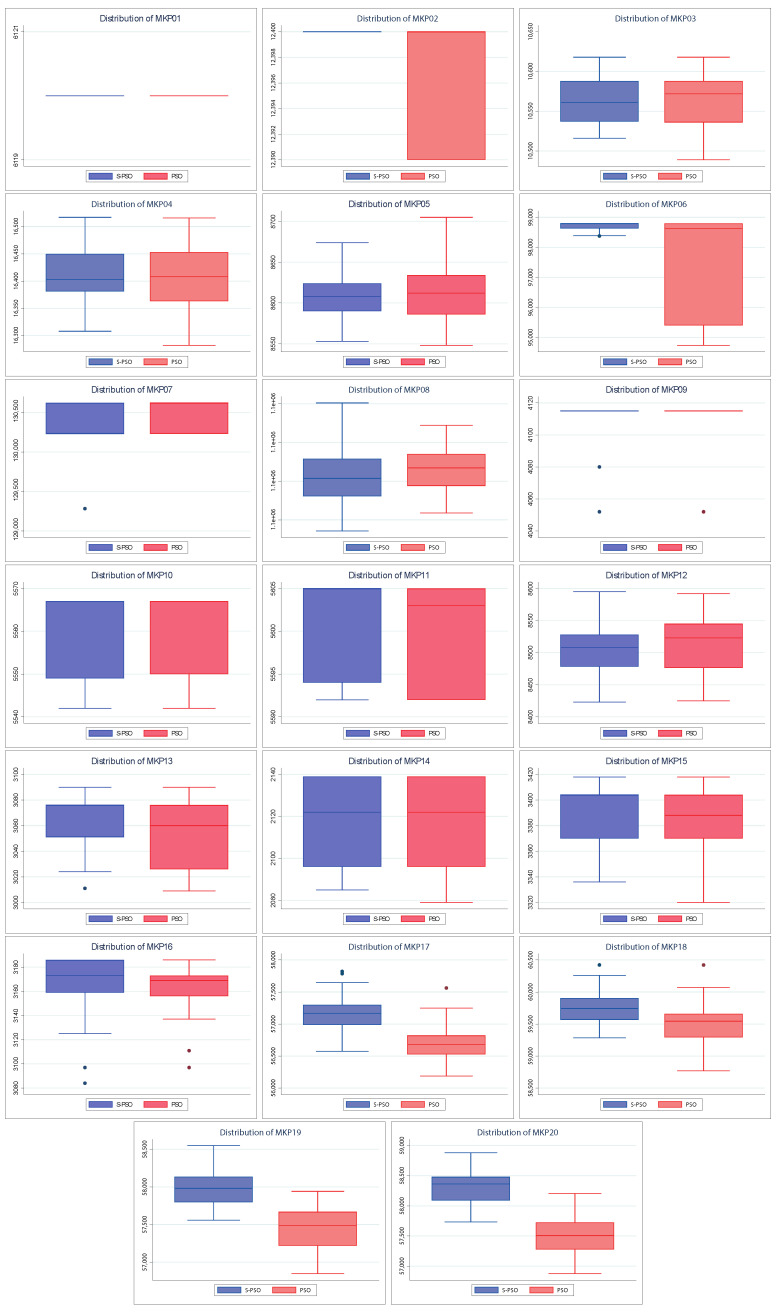
Distribution charts of PSO vs. Shannon PSO.

Also, Figure 5 shows the dispersion of the values found by both algorithms. The above allows knowing how close the values found are to their respective medians and their quartiles and extreme values. A better spread is smaller in size (more compact) and/or has larger medians and tails. As with convergences, the spreads of the S–PSO algorithm are usually at least similar to those of PSO. For MKP instances 3, 4, 5, 8, 10, and 12, the S–PSO spread is slightly lower. In MKP instances 1 and 9, both algorithms have the same dispersion. For the remaining 12 instances, S–PSO has values with lower dispersion and/or higher median. Therefore, it is considered to have better performance.

**Figure 6:**
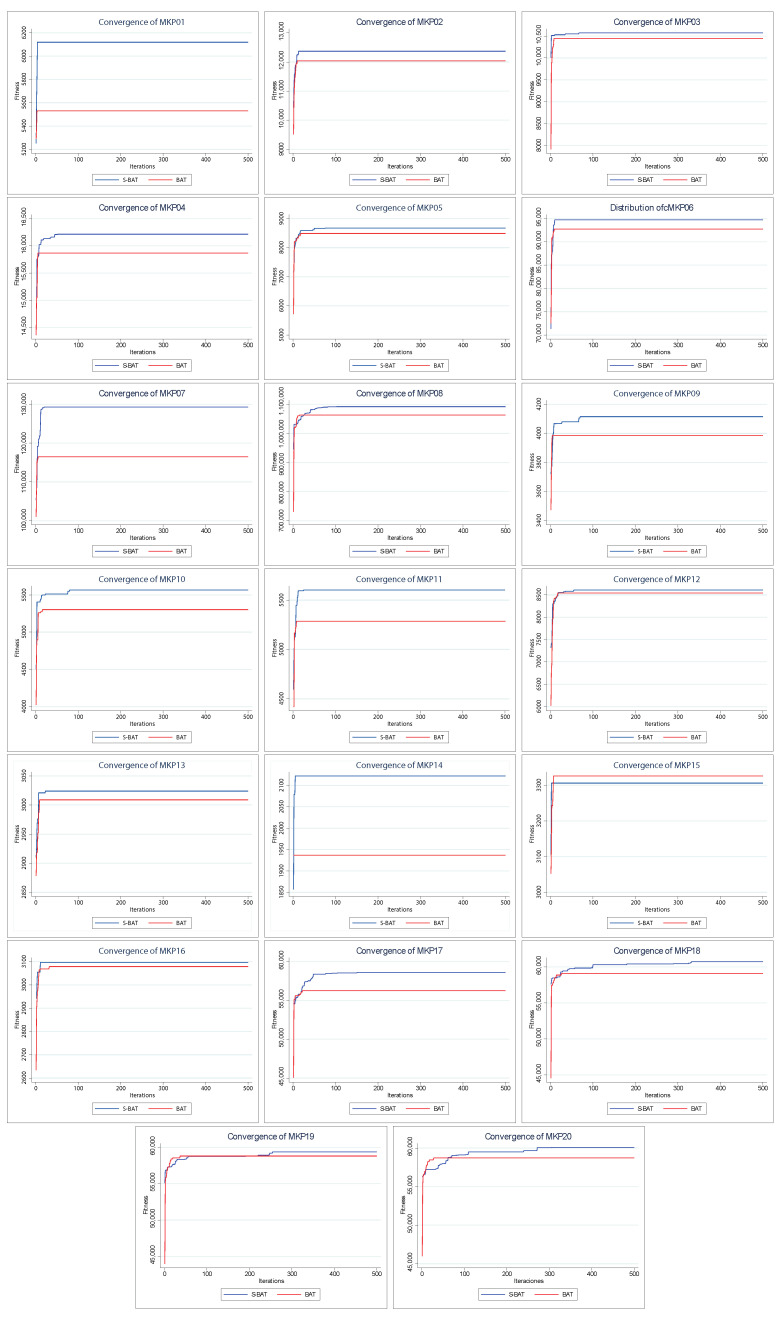
Convergence charts of BAT vs. Shannon BAT.

**Figure 7:**
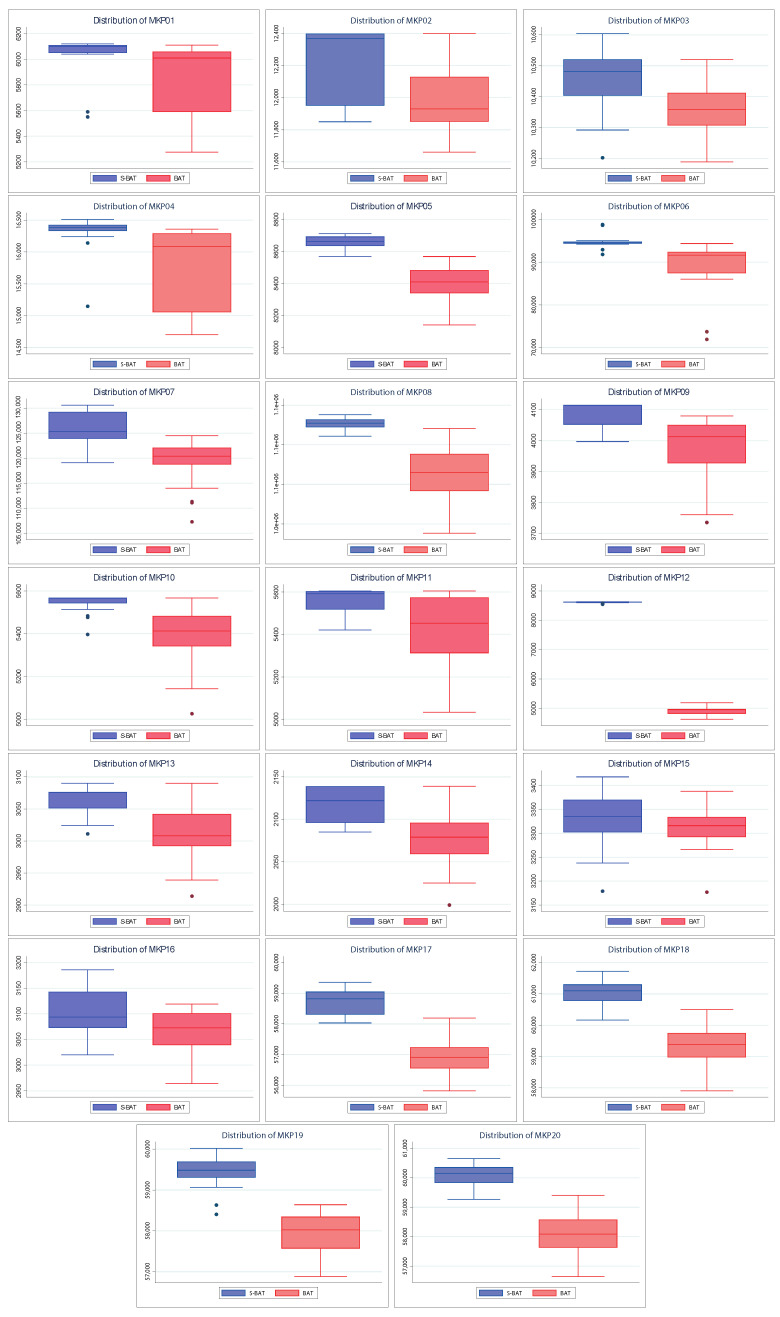
Distribution charts of BAT vs. Shannon BAT.

Figure 6 shows the convergence of the solutions found by BAT and S–BAT. In 19 of the 20 instances, S–BAT outperforms BAT. BAT is superior only in the MKP 15 instance. Both algorithms present similar values in early iterations, but S–BAT overlaps significantly as the execution progresses. On the other hand, Figure 7 shows the dispersion of the values found by both algorithms. In the 20 instances, the dispersion of the values found by S–BAT is smaller, presenting larger values and closer to their medians. Given the above, S–BAT has a significantly higher overall performance than BAT.

**Figure 8:**
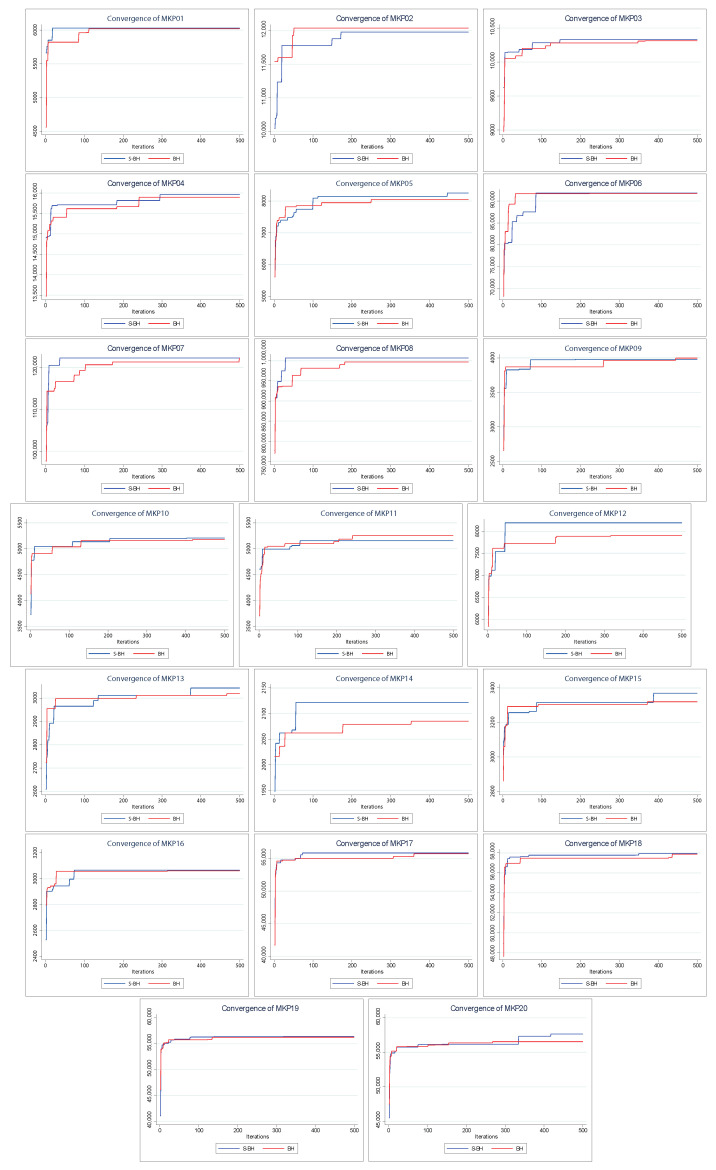
Convergence charts of BH vs. Shannon BH.

Figure 8 shows the convergences of the solutions found by BH and S–BH. For MKP instances 1, 3, 6, 16, 17, and 18, both algorithms present similar final values, with S–BH slightly higher. Only in three instances (MKP 2, 9, and 11) is the native BH algorithm slightly superior to S–BH. In the remaining eleven instances S–BH presents values significantly higher than BH.

**Figure 9:**
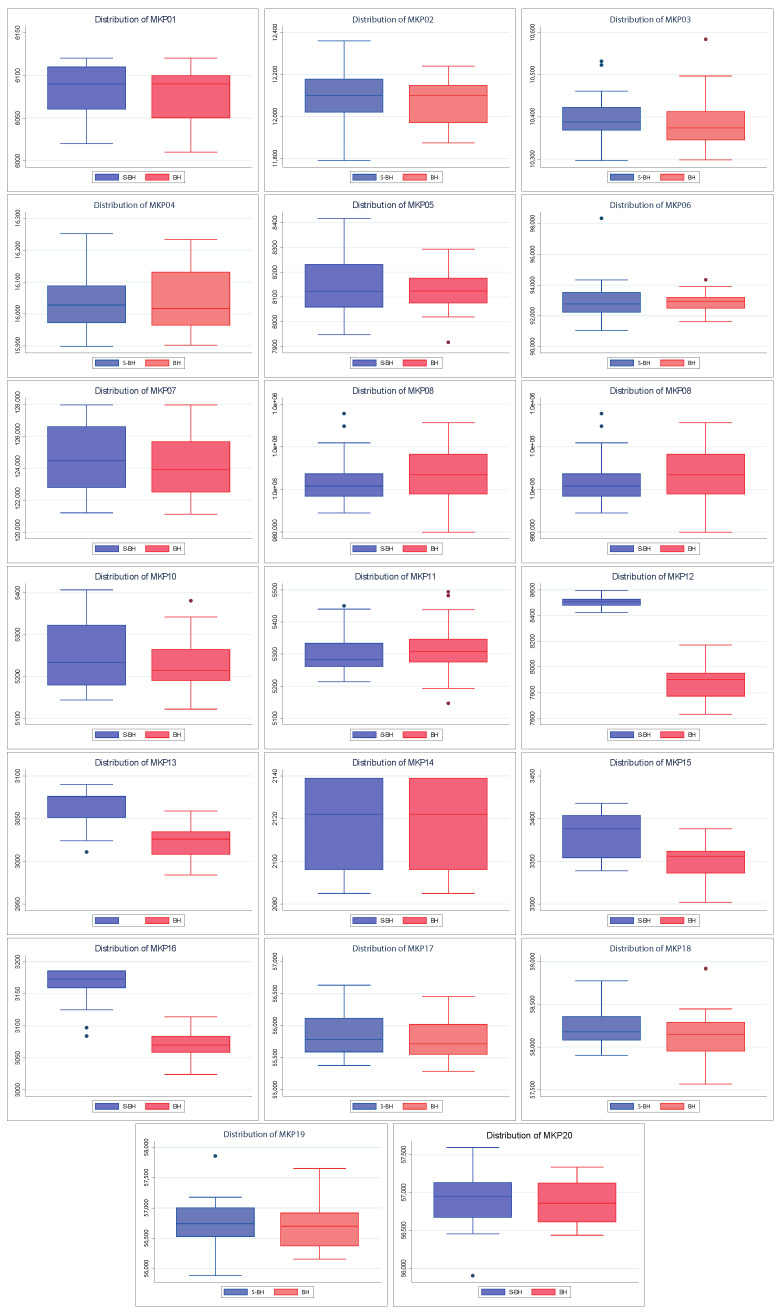
Distribution charts of BH vs. Shannon BH.

Furthermore, Figure 9 shows the dispersion of the values found by both algorithms. Because the behavior of these algorithms is more similar to each other than the previously seen ones (PSO and BAT), it is necessary to highlight that our performance approach is based on the largest values (maximization problem). Given the above, considering the MKP instances 1, 2, 5, 9, 17, 18, 19, and 20, we can say that S–BH has a better dispersion than BH. The above is because it has a median slightly higher than BH or larger extreme values. For MKP 6, 8, and 11 instances, native BH has superior performance for similar reasons. In the MKP 14 instance, both algorithms have the same dispersion. For the remaining eight instances, S–BH has a significantly higher dispersion in quality.

Finally, considering everything mentioned above, we can say that approximately half of the S–BH instances have at least a dispersion equal to or higher (quality) than that of BH. Therefore, it is considered to have better performance.

## 7. Statistical Analysis

To evidence a statistical significance between the native bio-inspired algorithm and its improved version by the Shannon strategy, we perform a robust analysis that includes normality assessment and contrast of hypotheses to determine if the samples come or not from an equidistributed sequence. Firstly, Shapiro–Wilk is required to study the independence of samples. It determines if observations (runs per instance) draw a Gaussian distribution. Then, we establish $H_{0}$ as samples follow a normal distribution. Therefore, $H_{1}$ assumes the opposite. The traditional limit of *p*-value is 0.05, for which results under this threshold state the test is said to be significant ($H_{0}$ rejects). Table 6 shows *p*-values obtained by native algorithms and their enhanced versions for each instance. Note that ∼0 indicates a small *p*-value near 0, and the hyphen means the test was not significant.

About 63% of the results confirm that the samples do not follow a normal distribution, so we decide to employ the non-parametric test Mann—Whitney—Wilcoxon. The idea behind this test is the following: if the two compared samples come from the same population, by joining all the observations and ordering them from smallest to largest, it would be expected that the observations of one and the other sample would be randomly interspersed [61].

To develop the test, we assume $H_{0}$ as the null hypothesis that affirms native methods generate better (smaller) values than their versions improved by the Shannon entropy. Thus, $H_{1}$ suggests otherwise. Table 7 exposes results of contrasts. Again, we use 0.05 as the upper threshold for *p*-values and smaller values allow us to reject $H_{0}$ and, therefore, assume $H_{1}$ as true. To detail the results obtained by the test, we deployed more significant digits, and we applied hyphens when the test was not significant.

Finally, and consistent with the previous results, the test strongly establishes that the bat optimizer is the bio–inspired method that benefits the most from the Shannon strategy (see Figure 6 and Figure 7). The robustness of this test is also evident with PSO and BH. We can see that the best results are adjusted to those already shown. For example, S–PSO on MKP02 and MKP06 instances is noticeably better than its native version (see Figure 4 and Figure 5). Similarly, S–BH on MKP12, MKP13, MKP15, and MKP16 instances performs better than the original version (see Figure 8 and Figure 9).

## 8. Conclusions

Despite the efficiency shown over the last few years, bio-inspired algorithms tend to stagnate in local optimal when facing complex optimization problems. During iterations, one or more solutions are not modified; therefore, resources are spent without obtaining improvements. Various methods, such as Random Walk, Levy Flight, and Roulette Wheel, use random diversification components to prevent this problem. This work proposes a new exploration strategy using the Shannon entropy as a movement operator on three swarm bio-inspired algorithms: particle swarm optimization, bat optimization, and black hole algorithm. The mission of this component is first to recognize stagnated solutions by applying information given by the solving process and then provide a policy to explore new promising zones. To evidence the reached performances by three optimization methods, we solve twenty instances of the 0/1 multidimensional knapsack problem, which is a variation of the well–known traditional optimization problem. Regarding the solving time, the results show that including an additional component increases the required time to reach the best solutions. However, in terms of accuracy to achieve optimal solutions, there is no doubt that this component significantly improves the resolution process of metaheuristics. We performed a statistical study on the results to ensure this conjecture was correct. As samples are independent and do not follow a normal distribution, we employ the Wilcoxon—Mann—Whitney test, a non-parametric statistical evaluation, to contrast the null hypothesis that the means of two populations are equal. Effectively, the swarm intelligence methods improved by the Shannon entropy exhibit significantly better yields than their original versions.

In future work, we propose comparing this proposal against other entropy methods, such as linear, Rényiand, or Tsallis, because they work an occurrence probability similar to Shannon. On the other hand, this research opens a challenge to analyze data generated by metaheuristics when internal search mechanisms operate. For example, the local search, exploration, or exploitation processes can converge on common ground. If this information is used correctly, we can be in front of powerful self-improvement techniques. In this scenario, we can design data-driven optimization algorithms capable of solving the problem and self-managing to perform this resolution in the best possible way.

## Figures and Tables

**Table 1:** Instances of the Multidimensional Knapsack Problem.

Instance	Name	Best Known	Knapsacks	Objects
MKP01	-	6120	10	20
MKP02	-	12400	10	28
MKP03	-	10618	5	39
MKP04	-	16537	5	50
MKP05	SENTO2 [57,58,59]	8722	30	60
MKP06	WEING5 [57,58,59]	98796	2	28
MKP07	WEING6 [57,58,59]	130623	20	28
MKP08	WEING7 [57,58,59]	1095445	2	105
MKP09	WEISH03 [58,59]	4115	5	30
MKP10	WEISH07 [58,59]	5567	5	40
MKP11	WEISH08 [58,59]	5605	5	40
MKP12	WEISH17 [58,59]	8633	5	60
MKP13	PB1 [58,59]	3090	4	27
MKP14	PB5 [58,59]	2139	10	20
MKP15	HP1 [58,59]	3418	4	28
MKP16	HP2 [58,59]	3186	4	34
MKP17	-	unknown	5	100
MKP18	-	unknown	5	100
MKP19	-	unknown	5	100
MKP20	-	unknown	5	100

**Table 2:** Experimental results.

ID	(a) Number of Best Reached						(b) Minimum Solving Time						(c) Maximum Solving Time					
	PSO	S–PSO	BA	S–BAT	BH	S–BH	PSO	S–PSO	BA	S–BAT	BH	S–BH	PSO	S–PSO	BA	S–BAT	BH	S–BH
MKP01	30	30	0	25	4	4	62	93	35	60	121	209	82	128	93	104	149	251
MKP02	20	28	1	9	0	0	64	99	30	46	251	82	78	127	100	79	95	104
MKP03	1	3	0	0	0	0	81	128	36	81	107	114	96	178	82	2821	131	137
MKP04	0	0	0	0	0	0	127	188	53	128	157	187	141	237	133	276	205	225
MKP05	0	0	0	0	0	0	412	421	8520	7503	13,827	13,649	517	535	162,779	60,468	18,624	17,446
MKP06	23	26	0	4	0	0	143	154	495	1099	2490	2536	180	203	1694	4872	2918	2772
MKP07	18	15	0	4	0	0	81	90	53	123	244	255	114	113	155	243	321	314
MKP08	0	0	0	0	0	0	120	159	57	109	110	150	148	199	120	174	145	191
MKP09	25	25	0	17	1	1	342	513	15,036	69,286	74,270	66,888	484	660	75,748	297,611	86,125	76,488
MKP10	23	23	2	16	0	0	559	456	12,909	32,863	41,213	38,791	456	696	323,688	213,580	53,025	47,676
MKP11	13	18	1	7	0	0	271	408	4639	6238	15,006	13,445	309	504	42,215	32,245	21,913	15,735
MKP12	0	0	0	7	0	0	304	216	240,867	821	2836	304	216	393	14,146,516	1955	3476	393
MKP13	1	4	1	4	0	4	51	89	35	99	82	89	76	131	101	142	100	131
MKP14	8	8	1	6	8	8	110	154	106	667	500	122	148	153	303	306	612	153
MKP15	6	7	0	1	0	3	52	90	54	49	77	87	75	133	177	123	110	162
MKP16	8	10	0	2	0	10	114	180	184	401	660	180	144	238	487	1322	795	238
MKP17	0	0	0	0	0	0	131	159	97	954,304	122	177	167	198	191	78,392,197	158	226
MKP18	0	0	0	0	0	0	132	165	98	177	123	129	188	202	233	415	150	206
MKP19	0	0	0	0	0	0	130	164	111	141	125	180	166	196	305	318	154	282
MKP20	0	0	0	0	0	0	112	155	105	167	127	184	174	203	467	541	161	214

**Table 3:** Experimental results of Shannon PSO against its native version.

ID	$Z_{\mathrm{opt}}$	Native PSO							Shannon PSO						
		$Z_{\max}$	${\mathrm{RPD}}_{\max}$	$Z_{\mathrm{med}}$	${\mathrm{RPD}}_{\mathrm{med}}$	$Z_{\mathrm{avg}}$	${\mathrm{RPD}}_{\mathrm{avg}}$	$Z_{\mathrm{sd}}$	$Z_{\max}$	${\mathrm{RPD}}_{\max}$	$Z_{\mathrm{med}}$	${\mathrm{RPD}}_{\mathrm{med}}$	$Z_{\mathrm{avg}}$	${\mathrm{RPD}}_{\mathrm{avg}}$	$Z_{\mathrm{sd}}$
MKP01	6120	6120	0.00	6120	0.00	6120	0.00	0.00	6120	0.00	6120	0.00	6120	0.00	0.00
MKP02	12,400	12,240	0.00	12,400	0.00	12,396.45	0.00	4.86	12,240	0.00	12,400	0.00	12,399.03	0.00	3.01
MKP03	10,618	10,618	0.00	10,572	0.00	10,562.70	0.00	31.92	10,618	0.00	10,561	0.00	10,565.51	0.00	31.29
MKP04	16,537	16,516	0.13	16,408	0.00	16,407.2	0.00	55.97	16,517	0.12	16,403	0.00	16,410.58	0.00	47.90
MKP05	8722	8674	0.55	8612	0.01	8609.90	0.01	27.63	8705	0.19	8608	0.01	8607.45	0.01	34.73
MKP06	98,796	98,796	0.00	98,796	0.00	97,473.16	0.01	1735.06	98,796	0.00	98,796	0.00	97,998.61	0.00	1503.18
MKP07	130,623	130,623	0.00	130,623	0.00	130,459.45	0.00	273.16	130,623	0.00	130,233	0.00	130,360.41	0.00	345.60
MKP08	1,095,445	1,074,459	1.92	1,063,435	0.02	1,063,110.38	0.02	5623.42	1,080,226	1.39	1,060,724	0.03	1061215.71	0.03	6497.72
MKP09	4115	4115	0.00	4115	0.00	4104.83	0.00	23.55	4115	0.00	4115	0.00	4105.74	0.00	21.94
MKP10	5567	5567	0.00	5567	0.00	5561.70	0.00	9.30	5567	0.00	5567	0.00	5561.67	0.00	9.34
MKP11	5605	5605	0.00	5603	0.00	5600.64	0.00	5.68	5605	0.00	5605	0.00	5601.45	0.00	5.43
MKP12	8633	8592	0.47	8523	0.01	8513.03	0.01	42.43	8595	0.44	8508	0.01	8507.32	0.01	42.17
MKP13	3090	3090	0.00	3060	0.00	3055.51	0.01	13.00	3090	0.00	3076	0.00	3063.06	0.00	22.04
MKP14	2139	2139	0.00	2122	0.00	2118.03	0.00	20.81	2139	0.00	2122	0.00	2118.70	0.00	17.56
MKP15	3418	3418	0.00	3388	0.00	3385.41	0.00	27.38	3418	0.00	3404	0.00	3382	0.01	26.40
MKP16	3186	3186	0.00	3173	0.00	3154.83	0.00	22.95	3186	0.00	3173	0.00	3165.03	0.00	25.08
MKP17	unknown	57,415	-	57,261	-	56,711.51	-	288.09	57,821	-	57,165	-	57,167.87	-	276.97
MKP18	unknown	60,421	-	59,544	-	59,505.48	-	342.39	60,423	-	59,743	-	59,766.22	-	268.25
MKP19	unknown	58,481	-	57,477	-	57,442.41	-	290.47	58,550	-	57,982	-	57,991.45	-	262.92
MKP20	unknown	58,880	-	58,325	-	58,321.22	-	338.59	59,021	-	58,363	-	58,336.61	-	318.37

**Table 4:** Experimental results of Shannon BAT against its native version.

ID	$Z_{\mathrm{opt}}$	Native BAT							Shannon BAT						
		$Z_{\max}$	${\mathrm{RPD}}_{\max}$	$Z_{\mathrm{med}}$	${\mathrm{RPD}}_{\mathrm{med}}$	$Z_{\mathrm{avg}}$	${\mathrm{RPD}}_{\mathrm{avg}}$	$Z_{\mathrm{sd}}$	$Z_{\max}$	${\mathrm{RPD}}_{\max}$	$Z_{\mathrm{med}}$	${\mathrm{RPD}}_{\mathrm{med}}$	$Z_{\mathrm{avg}}$	${\mathrm{RPD}}_{\mathrm{avg}}$	$Z_{\mathrm{sd}}$
MKP01	6120	6110	0.16	6010	0.01	5904.04	0.03	0.00	6120	0.00	6100	0.00	6017.61	0.01	0.00
MKP02	12,400	12,240	0.00	11,930	0.03	11,984.35	0.03	183.74	12,240	0.00	12,370	0.00	12,253.87	0.01	177.02
MKP03	10618	10,520	0.92	10,359	0.02	10,359.96	0.02	4.79	10,604	0.13	10,481	0.01	10,462.77	0.01	4.50
MKP04	16,537	16357	1.09	16,088	0.02	15,836.74	0.04	22.34	16,511	0.16	16,382	0.00	16,302.12	0.01	27.41
MKP05	8722	8568	1.77	8410	0.03	8405.77	0.03	42.56	8711	0.13	8663	0.00	8657.03	0.00	41.11
MKP06	98,796	94,348	4.50	91,618	0.07	89,691.32	0.09	196.63	98,796	0.00	94,738	0.04	95,067.19	0.03	35.85
MKP07	130,623	124,530	4.66	120,399	0.07	11,9467.67	0.08	4368.55	130,623	0.00	125,360	0.04	125,990.06	0.03	3655.85
MKP08	1,095,445	1,088,227	0.66	1,066,018	0.02	106,5867.29	0.02	4676.20	1,095,206	0.02	1,090,905	0.00	1,090,574.74	0.00	109.14
MKP09	4115	4080	0.85	4013	0.02	3983.96	0.03	56.62	4115	0.00	4115	0.00	4084.16	0.00	43.97
MKP10	5567	5567	0.00	5412	0.02	5398	0.03	1358.46	5567	0.00	5567	0.00	5545.80	0.00	224.29
MKP11	5605	5605	0.00	5452	0.02	5425.70	0.03	63.94	5605	0.00	5592	0.00	5557.51	0.00	20.02
MKP12	8633	8633	0.00	8410	0.02	8404.83	0.02	86.32	8633	0.00	8619	0.00	8612.48	0.00	41.79
MKP13	3090	3090	0.00	3008	0.02	3006	0.02	52.02	3090	0.00	3076	0.00	3063.06	0.00	52.16
MKP14	2139	2139	0.00	2079	0.02	2075.96	0.02	49.58	2139	0.00	2085	0.02	2097.74	0.01	56.23
MKP15	3418	3388	0.88	3316	0.02	3314.12	0.03	83.68	3418	0.00	3335	0.02	3330.09	0.02	92.92
MKP16	3186	3119	2.10	3073	0.02	3066.38	0.03	11210.30	3186	0.00	3094	0.02	3101.03	0.02	2991.02
MKP17	unknown	58,192	-	56,908	-	56,948.19	-	101.16	42,406	-	58,821	-	58,749.51	-	55.13
MKP18	unknown	60,502	-	59,380	-	59,374.67	-	92.26	60,423	-	61,098	-	61,041.671	-	56.33
MKP19	unknown	58,639	-	58,025	-	57,967.83	-	82.14	60,018	-	59,484	-	59,468.61	-	13.03
MKP20	unknown	59,402	-	58,089	-	58,048.77	-	40.61	60,654	-	60,151	-	60,105.80	-	13.47

**Table 5:** Experimental results of Shannon BH against its native version.

ID	$Z_{\mathrm{opt}}$	Native BH							Shannon BH						
		$Z_{\max}$	${\mathrm{RPD}}_{\max}$	$Z_{\mathrm{med}}$	${\mathrm{RPD}}_{\mathrm{med}}$	$Z_{\mathrm{avg}}$	${\mathrm{RPD}}_{\mathrm{avg}}$	$Z_{\mathrm{sd}}$	$Z_{\max}$	${\mathrm{RPD}}_{\max}$	$Z_{\mathrm{med}}$	${\mathrm{RPD}}_{\mathrm{med}}$	$Z_{\mathrm{avg}}$	${\mathrm{RPD}}_{\mathrm{avg}}$	$Z_{\mathrm{sd}}$
MKP01	6120	6110	0.00	6090	0.00	6083.22	0.00	30.48	6120	0.00	6090	0.00	6083.22	0.00	31.82
MKP02	12,400	12,240	1.29	12,100	0.02	12,084.03	0.02	98.62	12,360	0.32	12,100	0.02	12,091.77	0.02	118.87
MKP03	10,618	10,584	0.32	10,374	0.02	10,385.54	0.02	57.29	10,532	0.81	10,388	0.02	10,394.45	0.02	52.43
MKP04	16,537	16,234	1.83	16,017	0.03	16,046.80	0.02	97.87	16,252	1.72	16,028	0.03	16,038.03	0.03	84.18
MKP05	8722	8293	4.92	8124	0.06	8128.12	0.06	76.63	8417	3.50	8123	0.06	8140.32	0.06	113.49
MKP06	98,796	94,348	4.50	92,942	0.05	92,906.19	0.05	644.72	98,346	0.46	92,777	0.06	92,927.83	0.05	1330.35
MKP07	130,623	127,943	2.05	123,910	0.05	124,179.48	0.04	1937.12	127,953	2.04	124,462	0.04	124,476.41	0.04	2036.33
MKP08	1,095,445	998,864	8.82	1,006,919	0.08	1,007,945.67	0.07	12,822.05	999,278	8.78	1,001,572	0.08	1,004,224.58	0.08	10,919.46
MKP09	4115	4115	0.00	4024	0.02	4014.70	0.02	63.56	4115	0.00	4024	0.02	4030.93	0.02	50.38
MKP10	5567	5381	3.34	5214	0.06	5227.35	0.06	61.07	5407	2.87	5233	0.05	5251.25	0.05	81.32
MKP11	5605	5494	1.98	5308	0.05	5317.06	0.05	76.91	5450	2.77	5282	0.05	5301.03	0.05	62.07
MKP12	8633	8170	5.36	7902	0.08	7891.19	0.08	129.37	8595	0.44	8508	0.01	8507.32	0.01	42.17
MKP13	3090	3059	1.00	3026	0.02	3024.16	0.02	19.43	3090	0.00	3076	0.00	3063.06	0.00	22.04
MKP14	2139	2139	0.00	2122	0.00	2115.48	0.01	19.06	2139	0.00	2122	0.00	2118.70	0.00	17.56
MKP15	3418	3388	0.88	3356	0.01	3351.22	0.01	20.72	3418	0.00	3388	0.00	3382	0.01	23.39
MKP16	3186	3114	2.26	3070	0.03	3071.96	0.03	20.53	3186	0.00	3173	0.00	3165.03	0.00	25.08
MKP17	unknown	56,455	-	55,719	-	55,784.29	-	303.38	56,633	-	55,784	-	55,865.22	-	340.99
MKP18	unknown	58,921	-	58,149	-	58,132.38	-	264.85	59,097	-	58,102	-	58,192.51	-	342.95
MKP19	unknown	57,653	-	56,699	-	56,681.19	-	341.39	57,859	-	56,740	-	56,859	-	370.84
MKP20	unknown	57,337	-	56,861	-	56,859.54	-	280.80	57,597	-	56,948	-	56,908.64	-	345.66

**Table 6:** Test Shapiro–Wilk for native algorithms and their enhanced versions.

ID	Native Methods			Shannon Strategy		
	PSO	BAT	BH	S–PSO	S–BAT	S–BH
MKP01	∼0	–	0.00367	–	–	–
MKP02	–	0.04057	–	∼0	∼0	∼0
MKP03	∼0	–	0.00291	–	∼0	–
MKP04	∼0	∼0	∼0	0.00065	–	∼0
MKP05	–	–	∼0	0.00982	–	–
MKP06	∼0	0.00083	∼0	∼0	0.00072	∼0
MKP07	∼0	0.00065	∼0	∼0	0.0026	∼0
MKP08	∼0	–	∼0	0.0002	–	–
MKP09	–	0.0005	–	0.0082	∼0	∼0
MKP10	∼0	–	∼0	0.00712	0.00033	–
MKP11	∼0	0.00629	–	∼0	0.00037	∼0
MKP12	–	–	∼0	–	0.00001	–
MKP13	0.00036	0.0034	–	0.00036	0.00126	0.00126
MKP14	0.00011	∼0	0.00086	0.00011	0.00266	0.00015
MKP15	0.01802	–	0.00917	0.01802	–	0.00257
MKP16	0.00002	∼0	0.0054	0.00002	∼0	0.00002
MKP17	–	∼0	∼0	0.0006	0.03037	–
MKP18	∼0	∼0	–	0.00054	0.00076	∼0
MKP19	∼0	0.00658	∼0	0.06661	0.0012	∼0
MKP20	∼0	0.0054	∼0	0.0004	∼0	–

**Table 7:** Test Mann–Whitney–Wilcoxon for native algorithms against their enhanced versions.

ID	PSO vs. S–PSO	BAT vs. S–BAT	BH vs. S–BH
MKP01	–	2.9581384$\;\times \;10^{-9}$	–
MKP02	0.00014	3.9834857$\;\times \;10^{-7}$	–
MKP03	–	6.1540486$\;\times \;10^{-6}$	–
MKP04	–	2.9163915$\;\times \;10^{-11}$	–
MKP05	–	6.6611161$\;\times \;10^{-12}$	–
MKP06	0.00573	1.5107026$\;\times \;10^{-12}$	–
MKP07	–	9.5509242$\;\times \;10^{-8}$	–
MKP08	–	1.4444445$\;\times \;10^{-2}$	–
MKP09	–	4.7065594$\;\times \;10^{-9}$	–
MKP10	–	3.1609970$\;\times \;10^{-11}$	–
MKP11	–	4.9246126$\;\times \;10^{-6}$	–
MKP12	–	1.1318991$\;\times \;10^{-10}$	6.6650018$\;\times \;10^{-13}$
MKP13	0.00908663513	5.1131699$\;\times \;10^{-9}$	4.8034849$\;\times \;10^{-8}$
MKP14	–	0.0037256	–
MKP15	–	0.0208799	1.5990313$\;\times \;10^{-5}$
MKP16	–	0.0049714	5.8935079$\;\times \;10^{-12}$
MKP17	2.6426213$\;\times \;10^{-9}$	6.6571193$\;\times \;10^{-12}$	–
MKP18	1.4444445$\;\times \;10^{-12}$	1.0824563$\;\times \;10^{-12}$	–
MKP19	2.9162619$\;\times \;10^{-9}$	6.6688876$\;\times \;10^{-12}$	–
MKP20	1.0321732$\;\times \;10^{-11}$	7.3535622$\;\times \;10^{-12}$	–

## Data Availability

Not applicable.
